# Models of Polaron Transport in Inorganic and Hybrid Organic–Inorganic Titanium Oxides

**DOI:** 10.1021/acs.chemmater.3c00322

**Published:** 2023-04-18

**Authors:** Kazuki Morita, Matthias J. Golomb, Miguel Rivera, Aron Walsh

**Affiliations:** † Department of Materials, Imperial College London, London SW7 2AZ, United Kingdom; ‡ Department of Chemistry, University of Pennsylvania, Philadelphia, Pennsylvania 19104-6323, United States; ¶ Department of Chemistry, University College London, London WC1H 0AJ, United Kingdom; § Department of Physics, Ewha Womans University, Seoul 03760, Korea

## Abstract

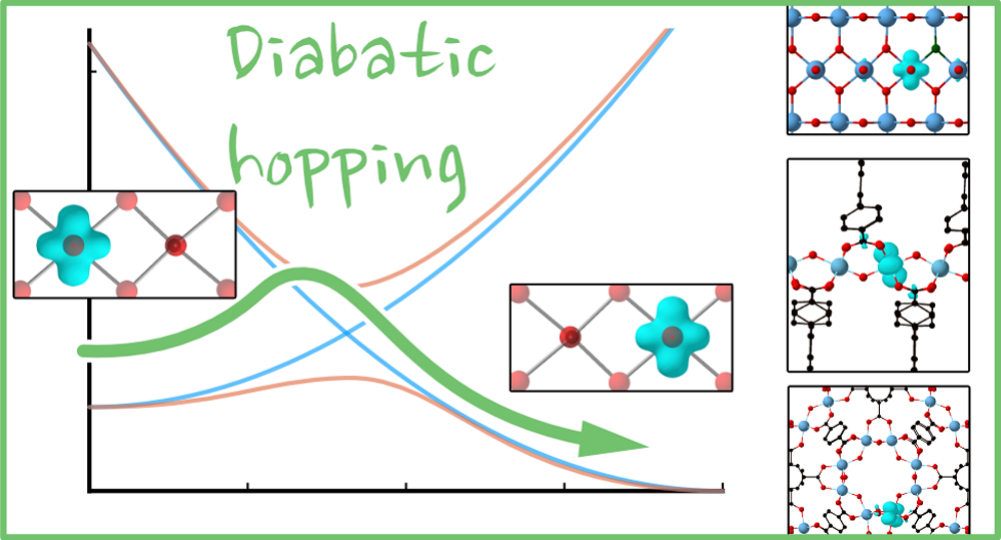

Polarons are a type of localized excess charge in materials and often form in transition metal oxides. The large effective mass and confined nature of polarons make them of fundamental interest for photochemical and electrochemical reactions. The most studied polaronic system is rutile TiO_2_ where electron addition results in small polaron formation through the reduction of Ti(IV) d^0^ to Ti(III) d^1^ centers. Using this model system, we perform a systematic analysis of the potential energy surface based on semiclassical Marcus theory parametrized from the first-principles potential energy landscape. We show that F-doped TiO_2_ only binds polaron weakly with effective dielectric screening after the second nearest neighbor. To tailor the polaron transport, we compare TiO_2_ to two metal–organic frameworks (MOFs): MIL-125 and ACM-1. The choice of MOF ligands and connectivity of the TiO_6_ octahedra largely vary the shape of the diabatic potential energy surface and the polaron mobility. Our models are applicable to other polaronic materials.

## Introduction

**Figure 1:**
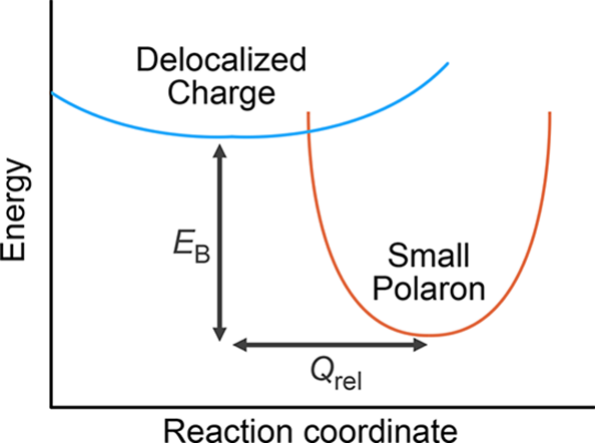
Schematic of small polaron formation. *E*_B_ is the binding energy of the polaron, and *Q*_rel_ is the structural reorganization with respect to the perfect crystal. The orange (blue) parabola represents polaron (delocalized) potential energy surface.

Localization of excess charge (electrons or holes) to form small polarons is a common feature of dielectric crystals in general and transition metal oxides in particular.^1−3^ There is a competition between the potential energy gained through polarization of the crystal from charge localization and the kinetic energy gained by delocalizing a charge within the extended valence or conduction bands. The localization of charge alters the chemical bonding (effective radii of the elements present) and is associated with a local structural distortion, which accompanies the charge as it moves through the crystal (Figure 1). Small polaron formation, with respect to a delocalized band electron, is enhanced by a large band effective mass and a strong low-frequency dielectric response.^4,5^ These features are found in TiO_2_, where the conduction band formed of the Ti(IV) d^0^ orbitals is combined with large Born effective charges that give rise to a large dielectric constant.^6,7^

The behavior of excess electrons and holes in TiO_2_ has been the subject of intense study owing to applications in photoelectrochemistry, photovoltaics, and catalysis.^8−12^ Although spatially localized, polarons can influence the optical absorption, reactivity, and electronic conductivity, making knowledge of them essential for materials optimization.^13−15^ Pristine crystals are diamagnetic due to the closed shell Ti(IV) d^0^ configuration. The one-electron reduction to form spin-polarized Ti(III) d^1^ centers allows for selective spectroscopic probes including electron paramagnetic resonance (EPR). For example, an EPR study of an undoped single crystal of rutile TiO_2_ by Halliburton and colleagues measured polaron trapping below 15 K, with a characteristic activation energy of just 24 meV.^16^ The polaron in TiO_2_ surfaces can also be directly observed by scanning tunnelling microscopy, making it an ideal test bed for characterization.^17,18^

While TiO_2_ has been the subject of various polaron-focused studies, theoretical studies on polarons in metal–organic frameworks (MOFs) are rare, often only looking at the stability of polarons in the framework calculated via cluster models.^19−21^ Similar structural motifs to TiO_2_ can be found in MOFs. For example, MIL-125 is an electrochromic crystal containing rings of corner-sharing octahedra connected by the 1,4-benzenedicarboxylate (bdc) ligand,^22^ while ACM-1 features continuous Ti–O chains with 4,4′,4″,4‴-(pyrene-1,3,6,8-tetrayl) tetrabenzoic acid (H4TBAPy) as a pillaring ligand.^23^ To the best of our knowledge, polaron transport studies on such periodic porous framework systems have not yet been performed. However, a number of MOFs based on Ti metal nodes with oxygen linkers have been synthesized and are known to host polaronic states upon electron or hole injection^24^ or photoexcitation,^25^ opening up the possibility to study polaron hopping from Ti to Ti sites via bridging oxygens comparable to those present in TiO_2_.

The present study concerns the behavior of polarons in TiO_6_ motifs within crystalline environments. In particular, we perform an in-depth analysis of the potential energy surfaces in the pristine and the F-doped rutile TiO_2_ and the prototype TiO_6_ containing MOFs MIL-125 and ACM-1. Analyzing the result of hybrid density functional theory (DFT) with semiclassical electron transport theory, we recover the diabatic potential energy surface, which includes key information on the polaron dynamics. We also highlight the possible avenue to designing materials with optimal polaron mobility for a given application.

## Methodology

### Potential Energy Surface for Polaron Hopping

Nudged elastic band (NEB) calculations were performed using DFT with a plane wave basis-set as implemented in VASP, which was modified by the VTST toolkit.^26−29^ The projector augmented-wave method was employed with Ti 3*d*^3^4*s*^1^ and O 2*s*^2^2*p*^4^ explicitly treated as valence electrons.^30^ The unit cell was expanded to 3 × 3 × 6 supercell, and the Brillouin zone was sampled at the Γ point. The plane wave expansion was cut off at 500 eV, and for the exchange-correlation functional, a screened hybrid exchange-correlation functional, HSE06, was employed.^31^ For NEB, seven intermediate states were considered between each polaron localization site.

To efficiently calculate MOFs with a large unit cell (and porous structure), NEB calculations were performed using the numerical atomic-centered basis set in FHI-Aims(32−36) via its integration into the ASE package.^37^ Polarons in MIL-125 were calculated in its conventional unit cell of 240 atoms, while ACM-1 was studied in a 2 × 1 × 1 supercell consisting of 156 atoms. Both systems were sampled at the Γ point, and the same HSE06 functional was used. Additionally, both frameworks were studied with the occupation matrix control method (details in the Supporting Information).^38^

### Polaron Transport

Small polaron transport within a crystal is commonly described in a quasi-static picture by employing Marcus theory.^39^ Within this theoretical framework, the polaron hopping from an initial to a final site is described as the transition between two corresponding diabatic states, which can yield a transfer rate following Landau–Zener theory. For the nonadiabatic approximation to hold, the electronic coupling between the ground and excited adiabatic states must be considerably weaker than the reorganization energy associated with the transfer.^40^

**Figure 2:**
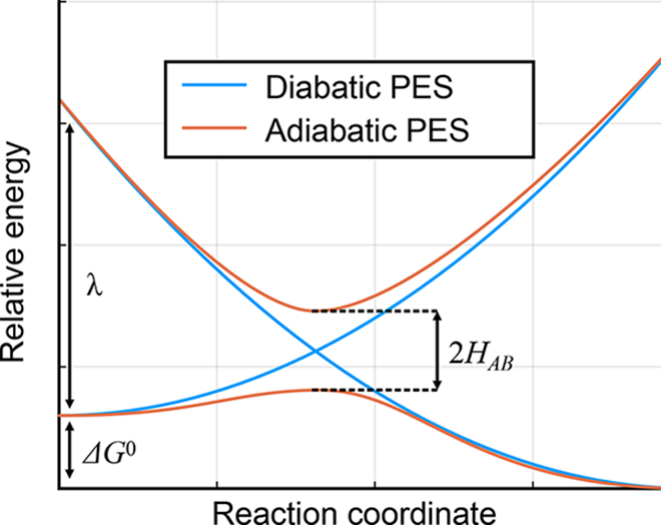
Schematic potential energy surface diagram showing the reorganization energy λ, change in Gibbs free energy Δ*G*^0^, and coupling energy *H*_*AB*_ in the Marcus theory formalism (eq 1).

The overall electron transfer rate can be described as
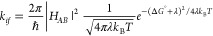
1where *H*_*AB*_ is the electronic coupling, *k*_B_ is the Boltzmann constant, λ is the reorganization energy, *T* is the temperature, and Δ*G*^0^ is the change in Gibbs free energy between the initial and final states. Obtaining λ depends on having an accurate representation of the diabatic potential energy surface of the final state, in the configuration of the initial state. Δ*G*^0^ depends on the minima at the initial and final states, which are identical between diabatic and adiabatic surfaces. Finally, coupling energy *H*_*AB*_ can be obtained by comparing the diabatic and adiabatic potential energy surfaces at the saddle point between initial and final states. These quantities are represented in Figure 2. The diabatic activation energy is often a telling indicator of the efficiency of Marcus transport and is related to the reorganization energy and electronic coupling as . The polaron mobility can then be obtained from the expression
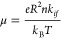
2where *e* is the elemental charge, *R* is the distance of hopping, and *n* is the number of neighbors. We have set *n* = 1 throughout this work.

If we could obtain a full diabatic *and* adiabatic representation of the potential energy surface, we would be able to readily calculate transfer rates for polarons. However, the ground state DFT calculations can only obtain one or the other, depending on the degree of localization of the polaron and the choice of DFT parameters. For instance, Deskins and Dupuis^41^ tuned the *U* parameter in GGA+*U* calculations of rutile and anatase TiO_2_ to enforce localized electronic density up to the saddle point within a linear interpolation. This has the advantage of obtaining a diabatic potential energy surface (PES), which would otherwise necessitate excited state calculations to characterize. Unfortunately, it comes at the cost of using an on-site Coulomb correction of *U* = 10 eV, where effective physical values for bulk transition metal oxides are between 3.1 and 6.4 eV.^42^ Deskins and Dupuis employ additional cluster model calculations based on the formalism of Reference (43) to obtain the missing electronic coupling. Furthermore, a direct comparison cannot be drawn with the analogous adiabatic PES obtained with a lower choice of *U*, due to the mismatch between levels of theory.

We chose to employ a hybrid exchange-correlation functional, obtaining localized polaron geometries, to produce a 1-D adiabatic PES via either linear interpolation or nudged elastic band. The diabatic potential energy surfaces are approximated as parabolas by sampling the energy values on the corresponding side of the saddle point. The values are then assigned weights according to a Boltzmann distribution, mirroring the increased anharmonicity away from the minimum, and the parabola is fitted by linear regression (details in the Supporting Information). The fitting procedure has been implemented in CarrierCapture, which also calculates Marcus transfer rates and charge carrier mobilities.^44^

## Results and Discussion

### Polaron in a Pristine Crystal

**Figure 3:**
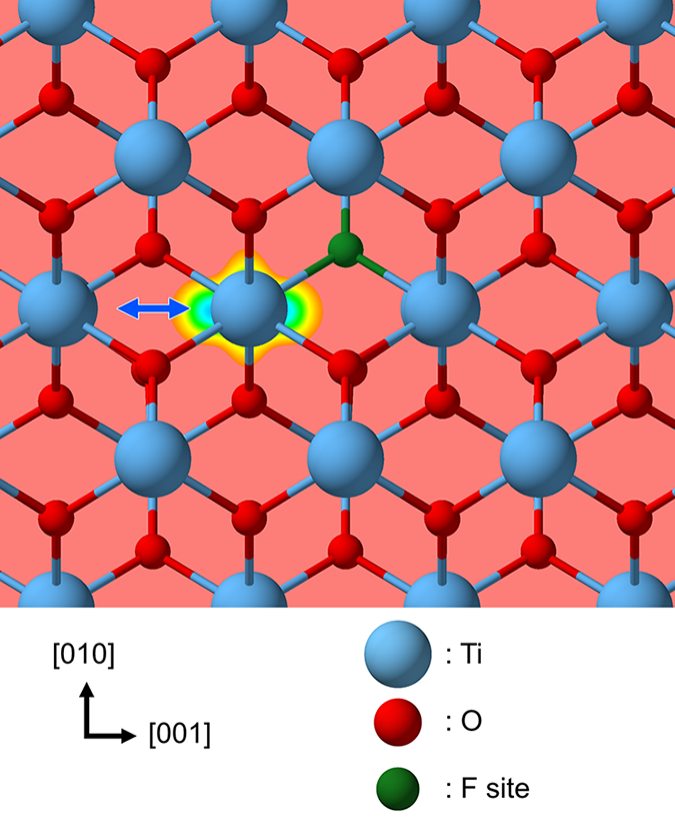
Crystal structure of rutile TiO_2_, highlighting the site for substitution with a fluorine atom. Here an effective electric dipole is formed between the F_O_^+^ and the polaron (e^–^). The electron density associated with the localized polaron state is plotted in the (100) plane. The blue arrow points to the [001] shortest distance hopping path.

The closest Ti–Ti distances in rutile TiO_2_ are in the [001] direction of the crystal. We, therefore, focus on this most efficient electron diffusion channel. To generate an electron polaron, in addition to simply adding an extra electron, we also considered explicit doping by substituting one neighboring oxygen with fluorine (i.e., F_O_), as depicted in Figure 3, which we will discuss in the next section.

The geometry of the Ti center and O nearest neighbors is depicted in Figure S1, which agrees with a previous computational study^45^ and within 0.2% error from an experimental value.^46^ We observe that a small stretch from the bulk geometry of the order of 0.01 Å in all six Ti–O bonds results in the localization of the electron polaron, with a magnetization of 0.75 μ_B_. These stretches agreed well with previous studies.^41,45^

**Figure 4:**
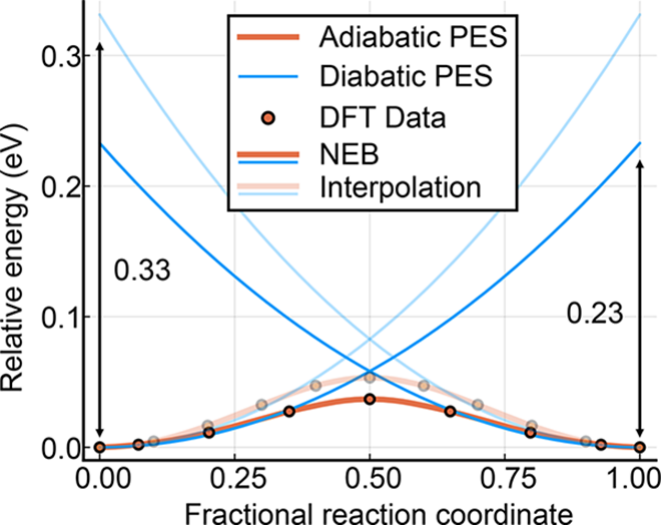
Potential energy surface of a nearest neighbor electron polaron hop in pristine TiO_2_, probed with linear interpolation and a transition state (NEB) search.

To investigate the polaron hopping rate, we probe its PES by two methods: linear interpolation and NEB. The two sets of data are presented in Figure 4. The energy barrier for the interpolated and the NEB pathways are 80 and 58 meV, and the reorganization energies are 0.33 and 0.23 eV, respectively. The lowering of the reorganization energy from the strained to the relaxed saddle point has a significant impact on the transport properties of the fitted Marcus model, as is reported in Table 1. The hopping rate increases from 9.64 × 10^11^ s^–1^ to 1.59 × 10^12^ s^–1^. The value obtained from NEB is closest to reality, where the polaron will adopt the least energetic path. Indeed, the diabatic activation energy for the interpolation is 80 meV, compared to 58 meV for NEB and 24 meV in EPR experiments.^16^ Experimentally, polaron mobilities have been reported in the range of 0.01–10.0 (cm^2^ V^–1^ s^–1^).^47−49^ Although the calculated value by NEB and linear interpolation falls into this range, the majority of mobility measurements are calculated from Hall measurements, where the effect from other carriers can be difficult to separate, so direct comparison requires care. Depending on the condition of the sample and experimental setup, treatment of surfaces, domain, defect scattering, phonon scattering, and finite temperature effects may be required.^47,49,50^ Furthermore, at higher temperatures, we expect a sizable contribution from a delocalized state that is only 0.15 eV above the ground state polaron structure, which would require a separate treatment.^51^

**Table 1:** Polaron Transport Data for the Pristine and Doped TiO_2_^a^

	Activation barrier (meV)	Coupling (meV)	Hopping rate (× 10^12^ s^–1^)	Mobility (cm^2^ V^–1^ s^–1^)
EPR^16^	24	-	-	-
Interpolation^b^	80	29	0.96	0.032
NEB^b^	58	21	1.59	0.053
Ti2–Ti1 NEB (trap)^c^	61	19	1.21	0.040
Ti1–Ti2 NEB (escape)^c^	41	19	2.94	0.098
Ti3–Ti2 NEB (trap)^c^	58	25	2.28	0.076
Ti2–Ti3 NEB (escape)^c^	64	25	1.68	0.056

a For the doped system, both the hop towards (trap) and away (escape) from the defect are reported, as illustrated in Figure 3.

b Pristine.

c Doped.

Deskins and Dupuis calculated a hopping rate of 7.65 × 10^11^ s^–1^ with an activation energy of 90 meV,^41^ which is close to our interpolated results. However, the increased energy barrier translates to a 1.15 eV reorganization energy, significantly larger than our 0.33 eV. This is compensated by an electronic coupling of 200 meV, an order of magnitude larger than those found by interpolation and NEB. The large increases in both reorganization energy and electronic coupling cancel out, leading to a modest difference of 1.99 s^–1^ with our own hopping rate from interpolated geometries. This demonstrates how small changes in the activation energy can be amplified in the hopping rate through the exponential dependence.

### Polaron in the F-Substituted Crystal

**Figure 5:**
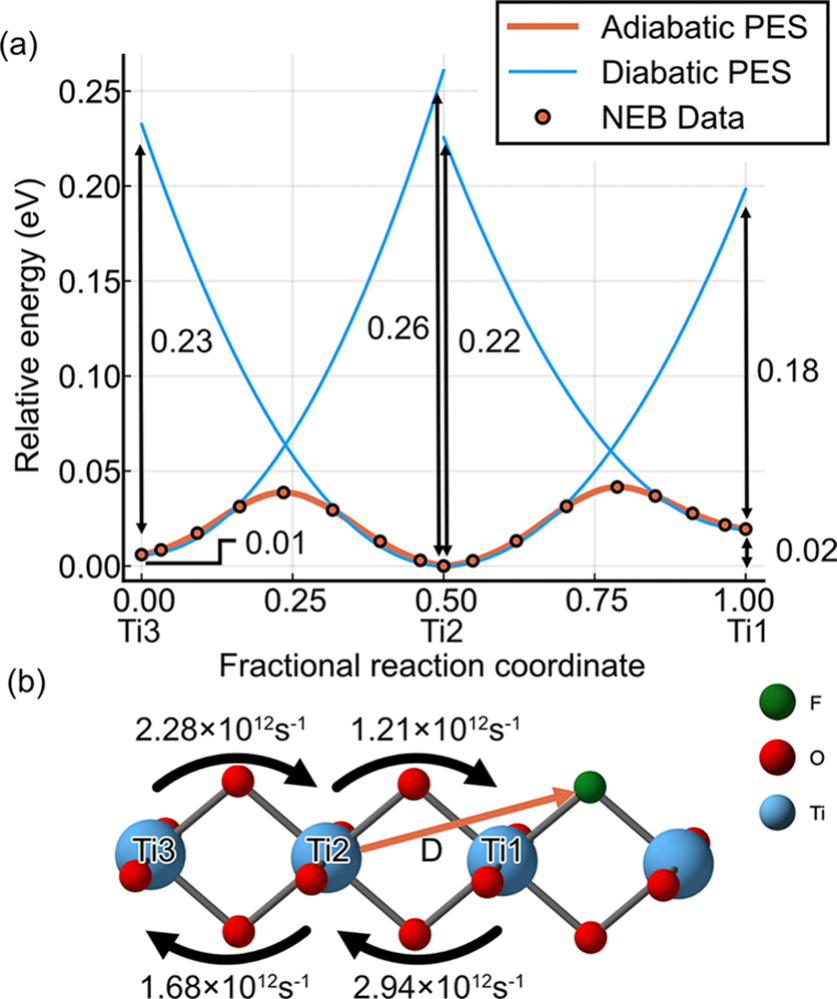
(a) Potential energy surface from a transition state (NEB) search for a polaron hop in the proximity of the F site in F-doped TiO_2_. 0 is away from the F site, and 1 is closer to it. The energy of the potential energy surface is taken relative to the lowest energy site. (b) Polaron mobility in the direction of trapping and escaping. D represents the dipole formed between a polaron at the Ti2 and F sites.

Next, we calculated the potential energy surface polaron hopping in three Ti site neighboring donors in F-doped rutile TiO_2_ (Figure 5 and Table 1). Here an effective dipole is created between the positively charged donor (F_O_^+^) and the negatively charged polaron (*e*^–^). As such, hopping to and away from the F site is no longer equivalent. The reorganization energy of 0.23 eV for the Ti2–Ti3 (escape) hop was the same as in the pristine system, indicating the negligible effect of F beyond this point due to effective dielectric screening by the crystal host. The polaron mobility was noticeably higher for Ti1–Ti2 (0.098 cm^2^ V^–1^ s^–1^) and Ti3–Ti2 hop (0.076 cm^2^ V^–1^ s^–1^). The low activation energy for the former and large coupling for the latter is the origin of the pronounced mobility.

Despite the electropositivity of the fluorine donor, the polaron is destabilized at its direct proximity, Ti1, by 20 meV, and the global minimum is located at the second nearest neighboring site, Ti2. This is in contrast to the case of a hydrogen donor where polarons are stable at the closest Ti-site.^52^ The difference originates from the large size of the fluorine dopant. The ionic radius of F^–^ is slightly larger than that of O^2–^, so F^–^ creates a strain field that elongates the bonds around itself with the cost of reducing the volume of the neighboring titanium octahedra (Figure S1). As the polaron prefers to elongate the surrounding bonds in TiO_2_, the strain field due to the F site competes with the stability of the polaron, and the polaron is pushed away from the F site. Yet, the interaction is not totally repulsive, and Ti2 is preferred over Ti3 due to the Coulombic interaction between the polaron and the F^–^. If we assume a fully classical picture, ionized fluorine dopant is in the −1 charge state, which is +1 relative to the −2 for oxygen in TiO_2_, and a polaron is −1, which therefore causes an attractive interaction. The actual difference was only 10 meV, so we expect reduced effective charge and a sizable screening effect from the surrounding polarizable crystal. The stabilization due to this effect is comparable to thermal energy at room temperature, so we expect that F only weakly binds the polaron and effectively ionizes at room temperature.

### Polarons in Metal–Organic Frameworks

**Figure 6:**
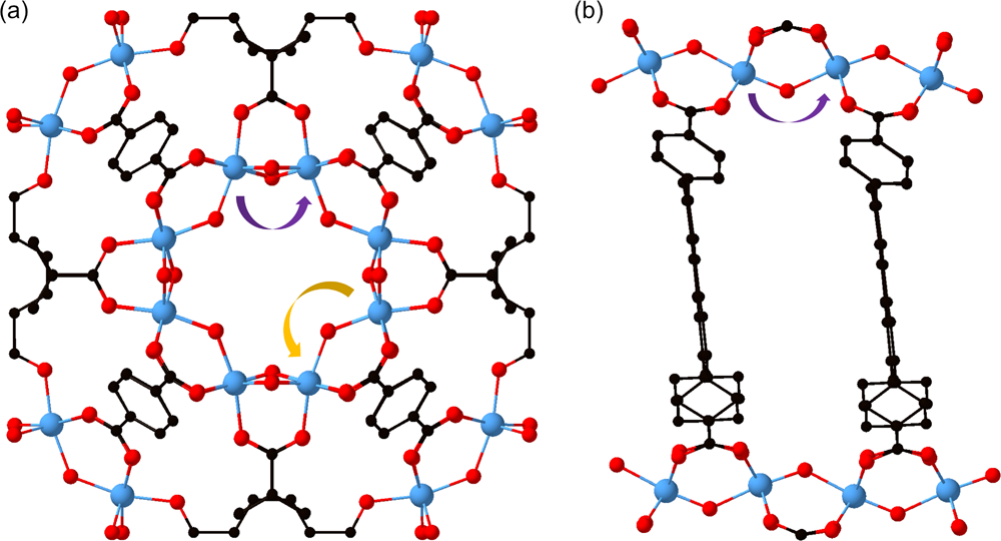
Crystal structures of (a) MIL-125 and (b) ACM-1. The arrows indicate the polaron hopping paths considered. For MIL-125, the purple and yellow arrows correspond to the short and the long hopping, respectively. The black atoms indicate the carbon atoms in the ligands.

Both MIL-125 and ACM-1 show local chemical environments similar to TiO_2_, where metal sites are bridged by oxygen (Figure 6). In the former, two distinct hopping processes can be identified, where one is via two bridging oxygens and the other is via a single bridging oxygen atom. Due to the differing Ti site distances, we call them “short” (2.67 Å) and “long” hop (3.67 Å), respectively. ACM-1 on the other hand only has one likely hopping process along a continuous chain of Ti sites bridged by single oxygen atoms (3.43 Å).

**Figure 7:**
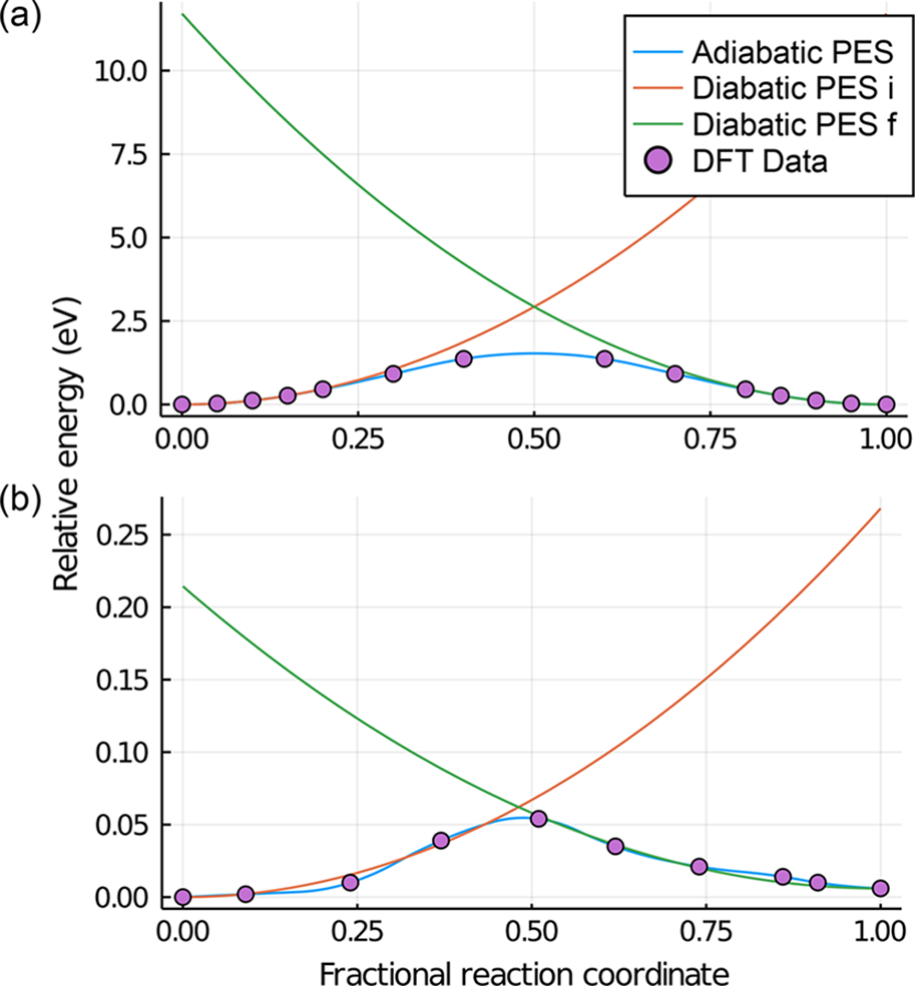
Potential energy surface of short hopping between neighboring Ti centers in MIL-125 calculated by (a) linear interpolation and (b) nudged elastic band transition state search. Diabatic PES i and f correspond to the initial and the final diabatic PES, respectively.

Relevant parameters such as hopping length, activation barrier, coupling, and reorganization energy are given in Table 2 for both linear interpolation and NEB. The potential energy surface for short hopping in MIL-125 is plotted in Figure 7. It is clear that linear interpolation fails to describe the hopping of a polaron in the considered MOFs, as it significantly overestimates all parameters compared to NEB. This suggests the non-negligible contribution of a nonlinear relaxation in the linkers/ligands. The results obtained via NEB however are reasonable and can be used for comparison with TiO_2_. Comparing these in Tables 2 and S1 between MIL-125 and TiO_2_, the short hop of MIL-125 had a comparable activation barrier (62 meV) but a smaller coupling of 8 meV. The long hop had comparable coupling energy (21 meV) but a higher activation barrier (91 meV). Both cases resulted in lower mobility. On the other hand for ACM-1, the activation barrier was actually smaller than TiO_2_; however, the resulting mobility was smaller (0.002 cm^2^ V^–1^ s^–1^) due to weak coupling of 3 meV. These results suggest the importance of having both a small activation barrier and a large coupling.

**Table 2:** Potential Energy Surface Profile Parameters of Polaron Hopping in MIL-125 and ACM-1^a^

		Hopping length (Å)	Activation barrier (meV)	Coupling (meV)	Reorganization energy (meV)
MIL-125 (short)	interpolation	2.70	2927	1396	11706
	NEB		62	8	208
MIL-125 (long)	interpolation	3.67	8081	3439	32544
	NEB		91	25	432
ACM-1	interpolation	3.43	280	69	1067
	NEB		48	3	114
TiO_2_	interpolation	2.94	80	29	330
	NEB		58	21	230

a Results for TiO_2_ are shown for comparison.

Based on chemical intuition that the TiO_6_ motif is similar across all systems, it is tempting to explain the trend in the activation barrier in terms of the hopping distance. Comparing the two hopping paths in MIL-125, it seems that the hopping barrier follows this trend. However, the activation barrier of the short hop in MIL-125 is higher than that of TiO_2_ while having a smaller hopping length, and ACM-1 has the smallest barrier overall despite its relatively large hopping length (3.43 Å). All this implies that the polaron itself is not the same across the systems and that the differences in polarization and localization must be taken into account to explain the resultant hopping behavior.

**Figure 8:**
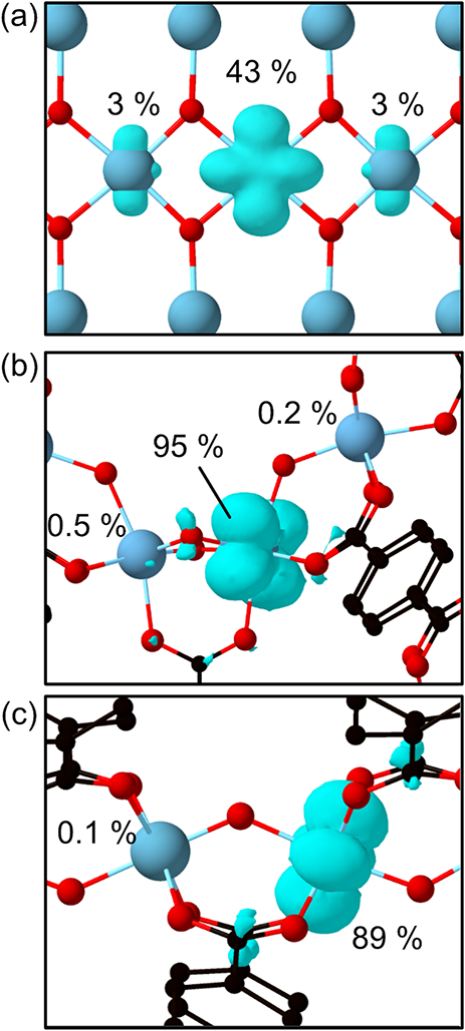
Electronic density distribution of Ti(III) polarons for (a) TiO_2_, (b) MIL-125, and (c) ACM-1. The percentage attributed to each Ti site is shown.

A stark difference is discerned when we plot the polaron density (spin density) at the PES minima (Figure 8). In TiO_2_, the antinode of the d-orbital wave function is pointing toward the neighboring Ti site, whereas in MOFs, the d-orbital is extending in the plane perpendicular to the direction of the short hopping. This points toward a different occupation of d-orbital levels arising from a change in crystal field splitting between the inorganic and hybrid materials. The symmetry mismatch is likely to have reduced the wave function overlap between the initial and the final site, leading to a decrease in electronic coupling. This is corroborated by our results, which show smaller couplings for the short hop in MIL-125 and in ACM-1, despite activation barriers comparable to those of TiO_2_. As a consequence, hopping rates in the studied MOFs are smaller, of the order of 10^11^ s^–1^ (MIL-125) and 10^9^ s^–1^ (ACM-1) (Table S1). It should be noted however that regardless of the largely d-orbital nature of the polaron at the PES minima, we were unable to reproduce the polaron behavior along the reaction pathway solely by modeling the occupation matrix of the d-orbitals, which suggests some contribution from coupling with the neighboring O 2p orbitals (Figure S3).

Further insight can be gained by studying the extension in space of a single polaron in the differing structures. We calculated the percentage of polaron density on the main Ti site using the Bader charge analysis in TiO_2_, MIL-125, and ACM-1, and it was 43%, 95%, and 89%, respectively.^53^ It is also visible in Figure 8 that the polaron in TiO_2_ has “tails” on the neighboring Ti sites in the [001] direction, which have also been reported previously.^54^ This points toward polarons in MOF building blocks localizing stronger than in inorganic materials, which is unsurprising due to the suppression of long-range polarization in such low-density nanoporous frameworks. From these results, we suggest that the strength of localization and the orientation of the polaron largely affect the activation barrier. The dimensionality of the TiO_6_ chain also plays a role as the strength of electron correlation changes with dimensionality.^55^ Indeed, we have previously shown that polarons in 2D TiO_6_ layers in Sr_3_Ti_2_O_7_ localize differently to TiO_2_.^56^ We, therefore, expect the isolated ring geometry in MIL-125 and isolated 1D chain geometry in ACM-1 itself to have the effect of strengthening the localization of polarons.

The above results highlight the diverse behavior of polarons accessible by changing the chemical environment of the TiO_6_ chain. MOFs are modular materials, and the ubiquitous choice of the ligand allows for various Ti–Ti distances, TiO_6_ orientations, and TiO_6_ chain topologies. Although beyond the scope of this study, just like in TiO_2_,^57^ intrinsic defects may obstruct the polaron transport in MOFs. The analysis in Figure 8 is possibly useful to screen MOFs for further materials discovery as the calculation does not require costly NEB calculations. We have used Bader charge analysis to decompose the polaron charges, but other means of volume decomposition such as Voronoi decomposition or simple integration within the Wigner–Seitz radius are likely to reproduce similar results. While a direct control of the d-orbital occupation matrix does not reproduce adiabatic barriers accurately, the values for diabatic transition state energies and reorganization energies appear to be promising, suggesting that it might be a useful tool for low-cost screening approaches. If the lattice relaxation away from the linearly interpolated reaction coordinates is small and does not qualitatively change the polaron hopping behavior, the ∼50 times smaller computational cost of linear interpolation can make it an efficient estimation tool. On the other hand, if one wants to quantitatively compare the initial and the final states, our method to extrapolate the diabatic PES from NEB adiabatic barriers is shown to work well for TiO_6_ motifs.

Finally, we want to review approximations made in this study and provide a possible avenue for future developments. Treatment of the one-dimensional reaction coordinate is a strong approximation, and when other phonon modes have a comparable electron–phonon coupling strength, they may modify the activation barrier, like in the case of “promoting modes” in nonradiative charge carrier transitions.^50^ The diabatic potential energy surface was used throughout this work; however, when the coupling energy is large, adiabatic treatment may suit the problem better. Polaron mobility depends on defect concentration.^47^ When defect concentration and species are known, we could perform similar analysis to that of F-doped TiO_2_ in this study to obtain the polaron mobility of the whole system. If the polaron is further delocalized to a Frölich polaron, mobility is likely to be explained better by models based on response function.^58^

## Conclusions

In this work, we have analyzed the formation of transport of Ti(III) polarons in different crystal environments. The diabatic polaron hopping behavior was extracted from an adiabatic first-principles potential energy surface. Our method does not restrict any exchange-correlation functional and allows the optimal choice to describe the polaron localization in correlated systems. We showed that F-doping in TiO_2_ is different from H-doping and weakly traps a polaron in the second-nearest neighbor site.

Through analysis of MOFs we found that the activation energy could vary largely depending on the connectivity of the TiO_6_ chain and the choice of ligands/linkers. The dynamics of polarons in MOFs will be further influenced by the long-range framework topologies, as well as structural defects that can be present in high concentrations. Our work does suggest that a wide range of carrier mobility will be accessible in MOFs through appropriate crystal engineering.

Linear interpolation qualitatively reproduces the nudged elastic band potential energy surface for TiO_2_, suggesting it to be a computationally inexpensive method to screen systems with smaller lattice relaxation. We also show that polaron density could be a descriptor to quantitatively understand the difficulty of hopping. Throughout this work, we focused on the electronic polaron in titanium oxides, but our approach is not restricted to these systems. Hole polarons are known to favor even stronger localization than electron counterparts, which suggests that our method is applicable to them. The validity in other host materials could be assessed by comparing the coupling energy with those reported in this work.

## Supporting Information Available

Details on the TiO_6_ geometry, potential energy surfaces, Boltzmann weighting, and matrix control method (PDF)

## Notes

The CarrierCapture package used in this work is available from https://doi.org/10.5281/zenodo.7828687.

## References

[ref1] Austin I.; Mott N. F. Polarons in Crystalline and Non-crystalline Materials. Adv. Phys. 1969, 18, 41–102. 10.1080/00018736900101267.

[ref2] Lany S. Semiconducting Transition Metal Oxides. J. Phys.: Condens. Matter 2015, 27, 283203 10.1088/0953-8984/27/28/283203. 26126022

[ref3] Franchini C.; Reticcioli M.; Setvin M.; Diebold U. Polarons In Materials. Nat. Rev. Mater. 2021, 6, 560–586. 10.1038/s41578-021-00289-w.

[ref4] Pekar S. I. Local Quantum States of Electrons in an Ideal Ion Crystal. J. Exp. Theor. Phys. 1946, 16, 341–348.

[ref5] Fröhlich H. Electrons in Lattice Fields. Adv. Phys. 1954, 3, 325–361. 10.1080/00018735400101213.

[ref6] Lee C.; Ghosez P.; Gonze X. Lattice Dynamics and Dielectric Properties of Incipient Ferroelectric TiO_2_ Rutile. Phys. Rev. B 1994, 50, 13379 10.1103/PhysRevB.50.13379. 9975530

[ref7] Di Valentin C.; Pacchioni G.; Selloni A. Reduced and n-type Doped TiO_2_: Nature of Ti^3+^ Species. J. Phys. Chem. C 2009, 113, 20543–20552. 10.1021/jp9061797.

[ref8] Grant F. A. Properties of Rutile (Titanium Dioxide). Rev. Mod. Phys. 1959, 31, 646–674. 10.1103/RevModPhys.31.646.

[ref9] Fujishima A.; Honda K.; et al. TiO_2_ Photoelectrochemistry and Photocatalysis. Nature 1972, 238, 37–38. 10.1038/238037a0. 12635268

[ref10] O’Regan B.; Grätzel M. A Low-Cost, High-Efficiency Solar Cell Based on Dye-Sensitized Colloidal TiO_2_ Films. Nature 1991, 353, 737–740. 10.1038/353737a0.

[ref11] Haruta M.; Kobayashi T.; Sano H.; Yamada N. Novel Gold Catalysts for the Oxidation of Carbon Monoxide at a Temperature Far Below 0. DEG. C. Chem. Lett. 1987, 16, 405–408. 10.1246/cl.1987.405.

[ref12] Scanlon D. O.; Dunnill C. W.; Buckeridge J.; Shevlin S. A.; Logsdail A. J.; Woodley S. M.; Catlow C. R. A.; Powell M. J.; Palgrave R. G.; Parkin I. P.; et al. Band alignment of rutile and anatase TiO_2_. Nat. Mater. 2013, 12, 798–801. 10.1038/nmat3697. 23832124

[ref13] Morgan B. J.; Watson G. W. Intrinsic n-type defect formation in TiO_2_: a comparison of rutile and anatase from GGA+ U calculations. J. Phys. Chem. C 2010, 114, 2321–2328. 10.1021/jp9088047.

[ref14] Iwaszuk A.; Nolan M. Charge compensation in trivalent cation doped bulk rutile TiO_2_. J. Phys.: Conden. Matter 2011, 23, 334207 10.1088/0953-8984/23/33/334207. 21813953

[ref15] Rettie A. J. E.; Chemelewski W. D.; Emin D.; Mullins C. B. Unravelling Small-Polaron Transport in Metal Oxide Photoelectrodes. J. Phys. Chem. Lett. 2016, 7, 471–479. 10.1021/acs.jpclett.5b02143. 26758715

[ref16] Yang S.; Brant A.; Giles N.; Halliburton L. Intrinsic Small Polarons in Rutile TiO_2_. Phys. Rev. B 2013, 87, 125201 10.1103/PhysRevB.87.125201.

[ref17] Henderson M. A.; Lyubinetsky I. Molecular-Level Insights into Photocatalysis from Scanning Probe Microscopy Studi es on TiO_2_ (110). Chem. Rev. 2013, 113, 4428–4455. 10.1021/cr300315m. 23488875

[ref18] Diebold U. The Surface Science of Titanium Dioxide. Surf. Sci. Rep. 2003, 48, 53–229. 10.1016/S0167-5729(02)00100-0.

[ref19] Ortega-Guerrero A.; Fumanal M.; Capano G.; Tavernelli I.; Smit B. Insights Into the Electronic Properties and Charge Transfer Mechanism of a Porphyrin Ruthenium-Based Metal–Organic Framework. Chem. Mater. 2020, 32, 4194–4204. 10.1021/acs.chemmater.0c00356.

[ref20] Patwardhan S.; Schatz G. C. Theoretical Investigation of Charge Transfer in Metal Organic Frameworks for Electrochemical Device Applications. J. Phys. Chem. C 2015, 119, 24238–24247. 10.1021/acs.jpcc.5b06065.

[ref21] Pratik S. M.; Gagliardi L.; Cramer C. J. Engineering Electrical Conductivity in Stable Zirconium-Based PCN-222 MOFs with Permanent Mesoporosity. Chem. Mater. 2020, 32, 6137–6149. 10.1021/acs.chemmater.0c01847.

[ref22] Dan-Hardi M.; Serre C.; Frot T.; Rozes L.; Maurin G.; Sanchez C.; Férey G. A New Photoactive Crystalline Highly Porous Titanium (IV) Dicarboxylate. J. Am. Chem. Soc. 2009, 131, 10857–10859. 10.1021/ja903726m. 19621926

[ref23] Cadiau A.; Kolobov N.; Srinivasan S.; Goesten M. G.; Haspel H.; Bavykina A. V.; Tchalala M. R.; Maity P.; Goryachev A.; Poryvaev A. S.; et al. A Titanium Metal-Organic Framework with Visible-Light-Responsive Photocatalytic Activity. Angew. Chem. 2020, 132, 13570–13574. 10.1002/ange.202000158. 32315516

[ref24] Capano G.; Ambrosio F.; Kampouri S.; Stylianou K. C.; Pasquarello A.; Smit B. On the Electronic and Optical Properties of Metal-Organic Frameworks: Case Study of MIL-125 and MIL-125-NH_2_. J. Phys. Chem. C 2020, 124, 4065–4072. 10.1021/acs.jpcc.9b09453.

[ref25] Nasalevich M. A.; Hendon C. H.; Santaclara J. G.; Svane K.; Van Der Linden B.; Veber S. L.; Fedin M. V.; Houtepen A. J.; Van Der Veen M. A.; Kapteijn F.; et al. Electronic origins of photocatalytic activity in d^0^ metal organic frameworks. Sci. Rep. 2016, 6, 23676 10.1038/srep23676. 27020767 PMC4810359

[ref26] Kresse G.; Furthmüller J. Efficient Iterative Schemes for Ab Initio Total-energy Calculations Using A Plane-wave Basis Set. Phys. Rev. B 1996, 54, 11169 10.1103/PhysRevB.54.11169. 9984901

[ref27] Kresse G.; Furthmüller J. Efficiency of Ab-initio Total Energy Calculations for Metals And Semiconductors Using A Plane-wave Basis Set. Comput. Mater. Sci. 1996, 6, 15–50. 10.1016/0927-0256(96)00008-0. 9984901

[ref28] Sheppard D.; Terrell R.; Henkelman G. Optimization Methods for Finding Minimum Energy Paths. J. Chem. Phys. 2008, 128, 134106 10.1063/1.2841941. 18397052

[ref29] Henkelman G.; Jonsson H. Improved Tangent Estimate in the Nudged Elastic Band Method for Finding Minimum Energy Paths and Saddle Points. J. Chem. Phys. 2000, 113, 9978–9985. 10.1063/1.1323224.

[ref30] Blöchl P. E. Projector Augmented-wave Method. Phys. Rev. B 1994, 50, 17953 10.1103/PhysRevB.50.17953. 9976227

[ref31] Heyd J.; Scuseria G. E.; Ernzerhof M. Hybrid Functionals Based on a Screened Coulomb Potential. J. Chem. Phys. 2003, 118, 8207–8215. 10.1063/1.1564060.

[ref32] Blum V.; Gehrke R.; Hanke F.; Havu P.; Havu V.; Ren X.; Reuter K.; Scheffler M. Ab Initio Molecular Simulations with Numeric Atom-centered Orbitals. Comput. Phys. Commun. 2009, 180, 2175–2196. 10.1016/j.cpc.2009.06.022.

[ref33] Havu V.; Blum V.; Havu P.; Scheffler M. Efficient Integration for All-electron Electronic Structure Calculation Using Numeric Basis Functions. J. Comput. Phys. 2009, 228, 8367–8379. 10.1016/j.jcp.2009.08.008.

[ref34] Ren X.; Rinke P.; Blum V.; Wieferink J.; Tkatchenko A.; Sanfilippo A.; Reuter K.; Scheffler M. Resolution-of-Identity Approach to Hartree-Fock, Hybrid Density Functionals, RPA, MP2 And *GW* With Numeric Atom-centered Orbital Basis Functions. New J. Phys. 2012, 14, 053020 10.1088/1367-2630/14/5/053020.

[ref35] Marek A.; Blum V.; Johanni R.; Havu V.; Lang B.; Auckenthaler T.; Heinecke A.; Bungartz H.-J.; Lederer H. The Elpa Library: Scalable Parallel Eigenvalue Solutions for Electronic Structure Theory And Computational Science. J. Phys.: Condens. Matter 2014, 26, 213201 10.1088/0953-8984/26/21/213201. 24786764

[ref36] Levchenko S. V.; Ren X.; Wieferink J.; Johanni R.; Rinke P.; Blum V.; Scheffler M. Hybrid Functionals for Large Periodic Systems in an All-Electron, Numeric Atom-Centered Basis Framework. Comput. Phys. Commun. 2015, 192, 60–69. 10.1016/j.cpc.2015.02.021.

[ref37] Hjorth Larsen A.; Jørgen Mortensen J.; Blomqvist J.; Castelli I. E.; Christensen R.; Dułak M.; Friis J.; Groves M. N.; Hammer B.; Hargus C.; et al. The Atomic Simulation Environment - A Python Library for Working with Atoms. J. Phys.: Condens. Matter 2017, 29, 273002 10.1088/1361-648X/aa680e. 28323250

[ref38] Kick M.; Reuter K.; Oberhofer H. Intricacies of DFT+U, Not Only in a Numeric Atom Centered Orbital Framework. J. Chem. Theor. Comp. 2019, 15, 1705–1718. 10.1021/acs.jctc.8b01211. 30735386

[ref39] Marcus R. A. On the Theory of Oxidation-Reduction Reactions Involving Electron Transfer. I. J. Chem. Phys. 1956, 24, 966–978. 10.1063/1.1742723.

[ref40] Oberhofer H.; Reuter K.; Blumberger J. Charge Transport In Molecular Materials: An Assessment Of Computational Methods. Chem. Rev. 2017, 117, 10319–10357. 10.1021/acs.chemrev.7b00086. 28644623

[ref41] Deskins N. A.; Dupuis M. Electron Transport via Polaron Hopping in Bulk TiO_2_: A Density Functional Theory Characterization. Phys. Rev. B: Condens. Matter Mater. Phys. 2007, 75, 195212 10.1103/PhysRevB.75.195212.

[ref42] Wang L.; Maxisch T.; Ceder G. Oxidation Energies of Transition Metal Oxides Within the GGA + U Framework. Phys. Rev. B 2006, 73, 195107 10.1103/PhysRevB.73.195107.

[ref43] Farazdel A.; Dupuis M.; Clementi E.; Aviram A. Electric Field Induced Intramolecular Electron Transfer In Spiro Π-Electron Systems And Their Suitability As Molecular Electronic Devices. A Theoretical Study. J. Am. Chem. Soc. 1990, 112, 4206–4214. 10.1021/ja00167a016.

[ref44] Kim S.; Hood S. N.; van Gerwen P.; Whalley L. D.; Walsh A. Carriercapture.Jl: Anharmonic Carrier Capture. JOSS 2020, 5, 2102 10.21105/joss.02102.

[ref45] Spreafico C.; VandeVondele J. The Nature of Excess Electrons in Anatase and Rutile from Hybrid DFT and RPA. Phys. Chem. Chem. Phys. 2014, 16, 26144–26152. 10.1039/C4CP03981E. 25360624

[ref46] Burdett J. K.; Hughbanks T.; Miller G. J.; Richardson J. W. Jr; Smith J. V. Structural-Electronic Relationships in Inorganic Solids: Powder Neutron Diffraction Studies of the Rutile and Anatase Polymorphs of Titanium Dioxide at 15 and 295 K. J. Am. Chem. Soc. 1987, 109, 3639–3646. 10.1021/ja00246a021.

[ref47] Yagi E.; Hasiguti R.; Aono M. Electronic conduction above 4 K of slightly reduced oxygen-deficient rutile TiO_2–*x*_. Phys. Rev. B 1996, 54, 7945–7956. 10.1103/PhysRevB.54.7945. 9984471

[ref48] Hendry E.; Wang F.; Shan J.; Heinz T. F.; Bonn M. Electron transport in TiO_2_ probed by THz time-domain spectroscopy. Phys. Rev. B 2004, 69, 081101 10.1103/PhysRevB.69.081101.

[ref49] Fujishima A.; Zhang X.; Tryk D. TiO_2_ photocatalysis and related surface phenomena. Surf. Sci. Rep. 2008, 63, 515–582. 10.1016/j.surfrep.2008.10.001.

[ref50] Stoneham A. M. Non-radiative transitions in semiconductors. Rep. Prog. Phys. 1981, 44, 1251–1295. 10.1088/0034-4885/44/12/001.

[ref51] Janotti A.; Franchini C.; Varley J. B.; Kresse G.; Van de Walle C. Dual Behavior of Excess Electrons in Rutile TiO_2_. Phys. Status Solidi RRL 2013, 7, 199–203. 10.1002/pssr.201206464.

[ref52] Deák P.; Aradi B.; Frauenheim T. Polaronic Effects in TiO_2_ Calculated by the HSE06 Hybrid Functional: Dopant Passivation by Carrier Self-trapping. Phys. Rev. B 2011, 83, 155207 10.1103/PhysRevB.83.155207.

[ref53] Henkelman G.; Arnaldsson A.; Jónsson H. A fast and robust algorithm for Bader decomposition of charge density. Comput. Mater. Sci. 2006, 36, 354–360. 10.1016/j.commatsci.2005.04.010.

[ref54] Elmaslmane A. R.; Watkins M. B.; McKenna K. P. First-Principles Modeling of Polaron Formation in TiO_2_ Polymorphs. J. Chem. Theory Comput. 2018, 14, 3740–3751. 10.1021/acs.jctc.8b00199. 29874462

[ref55] Pittalis S.; Räsänen E.; Helbig N.; Gross E. K. U. Exchange-Energy Functionals for Finite Two-Dimensional Systems. Phys. Rev. B 2007, 76, 235314 10.1103/PhysRevB.76.235314.

[ref56] Morita K.; Kumagai Y.; Oba F.; Walsh A. Switchable Electric Dipole from Polaron Localization in Dielectric Crystals. Phys. Rev. Lett. 2022, 129, 017601 10.1103/PhysRevLett.129.017601. 35841557

[ref57] Deák P.; Aradi B.; Frauenheim T. Quantitative Theory of the Oxygen Vacancy and Carrier Self-Trapping in Bulk TiO_2_. Phys. Rev. B 2012, 86, 195206 10.1103/PhysRevB.86.195206.

[ref58] Frost J. M. Calculating polaron mobility in halide perovskites. Phys. Rev. B 2017, 96, 195202 10.1103/PhysRevB.96.195202.

