# Supplementary material for: Models of Polaron Transport in Inorganic and Hybrid Organic–Inorganic Titanium Oxides
Source: Chem Mater. 2023 Apr 18;35(9):3652–9. doi: 10.1021/acs.chemmater.3c00322 (PMC10173375; doi:10.1021/acs.chemmater.3c00322)
Supplement: Supplementary file 1 — cm3c00322_si_001.pdf [file cm3c00322_si_001.pdf]

Supporting Information: Models of Polaron Transport in Inorganic and Hybrid Organic-Inorganic Titanium Oxides

Kazuki Morita,[†,‡] Matthias J. Golomb,[†] Miguel Rivera,[¶] and Aron Walsh[*,†,§]

†*Department of Materials, Imperial College London, London SW7 2AZ, UK*

‡*Department of Chemistry, University of Pennsylvania, Philadelphia, Pennsylvania 19104-6323, USA*

¶*Department of Chemistry, University College London, London WC1H 0AJ, UK*

§*Department of Physics, Ewha Womans University, Seoul 03760, Korea*

E-mail: a.walsh@imperial.ac.uk

Geometry around Ti in TiO_2

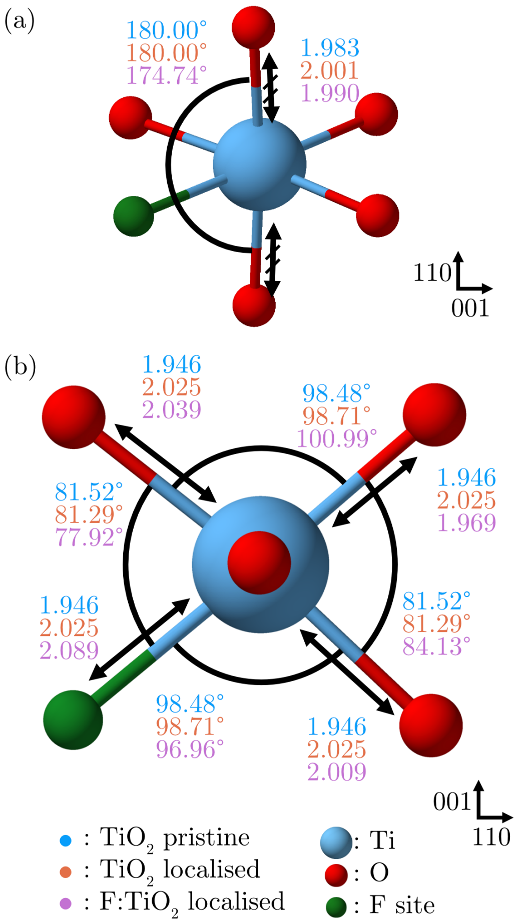

Figure S1: Geometry near the Ti site for bulk TiO_2 and the localised polaron for pristine and F-doped TiO_2.

Fitting the potential energy surface

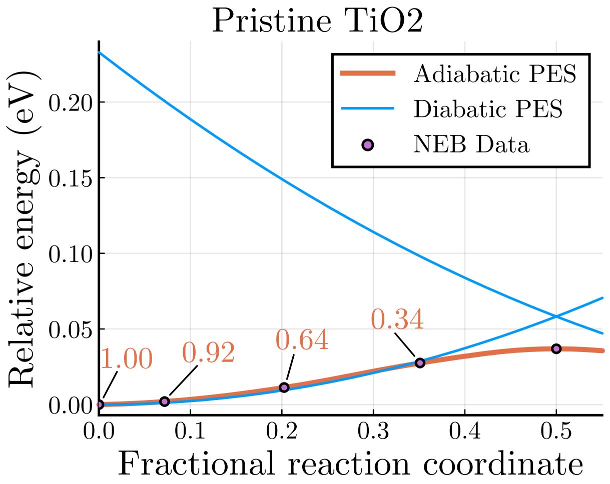

Figure S2: Potential energy surface of a polaron nearest neighbour hop. The Boltzman weights used in the diabatic potential fitting are included. The surface is symmetric about 0.5.

The harmonic potential was fitted with the following weight:

$$w = \exp\left(-\frac{E}{k_B T}\right), \tag{1}$$

where E is the relative energy with respect to a minima in the potential energy surface, k_B is the Boltzmann constant, and T is the temperature. The resulting weight is shown in Figure S2.

Potential energy surface of MOFs

Table S1: Nudged elastic band potential energy surface profile parameters of polaron hopping in MIL-125 and ACM-1. Results for TiO_2 are shown for comparison.

	Activation barrier (meV)	Coupling (meV)	Reorganisation energy (meV)	Hopping rate ($\times 10^{12}$ s^{-1})	Mobility ($cm^2V^{-1}s^{-1}$)
MIL-125 (short)	62	8	208	0.18	0.0060
MIL-125 (long)	91	25	432	0.46	0.015
ACM-1	48	3	114	0.077	0.002
TiO_2	58	21	230	1.59	0.053

Matrix control potential energy surface

Diabatic states are available when one can constrain the electron density to a particular site. This can be achieved in various ways,[S1] among which the occupation matrix control method has recently been implemented into the FHI-Aims package. Here, one can enforce a specific orbital of any atom to be occupied, similar to other constrained density functional theory (cDFT) methods, and promising results in common materials have been obtained.[S2] This method enables the calculation of the initial electronic state in the structural configuration of the final state and vice versa, allowing for a computationally efficient estimation of the diabatic parameters of the Marcus equation.

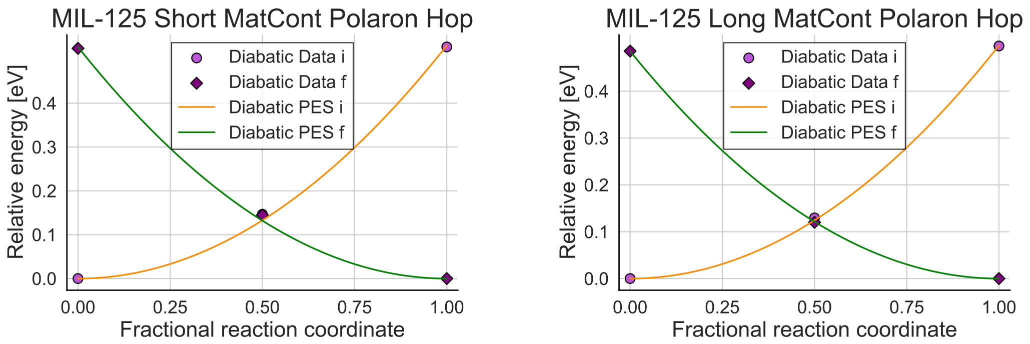

Figure S3: Matrix control potential energy surface of (left) short and (right) long hopping in MIL-125. Diabatic PES i and f corresponds to the initial and the final diabatic PES, respectively.

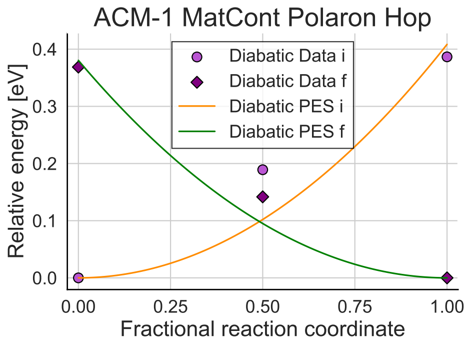

Figure S4: Matrix control potential energy surface of ACM-1. Diabatic PES i and f corresponds to the initial and the final diabatic PES, respectively.

Potential energy surfaces of MIL-125

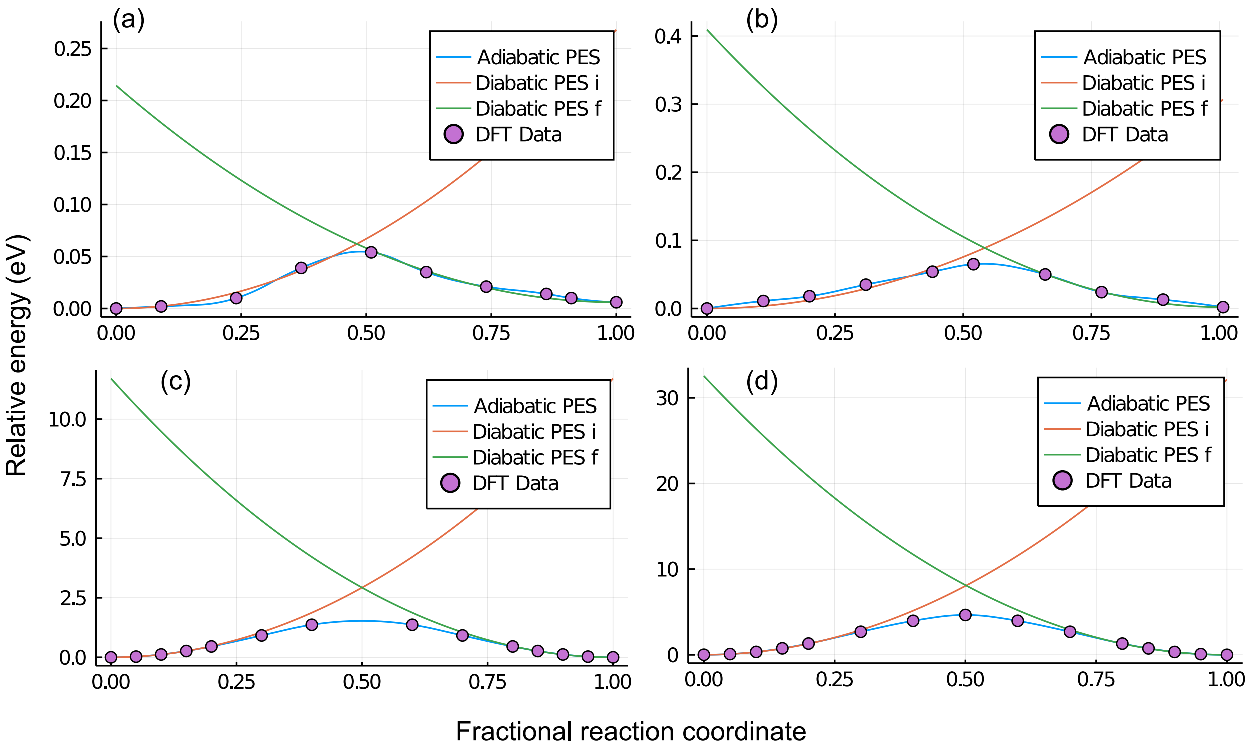

Figure S5: Linear interpolated potential energy surface of (a) short and (b) long hopping and nudged elastic band potential energy surface of (c) short and (d) long hopping in MIL-125. Diabatic PES i and f corresponds to the initial and the final diabatic PES, respectively.

Potential energy surfaces of ACM-1

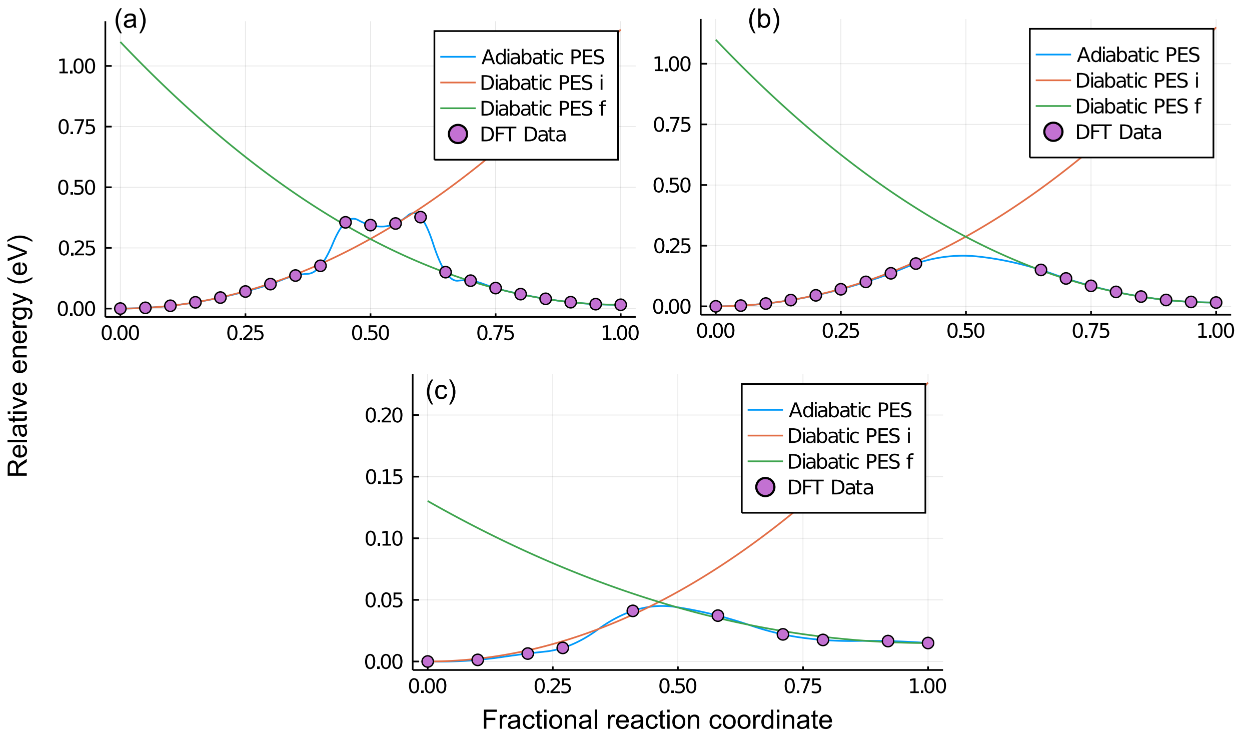

Figure S6: Linear interpolated potential energy surface of ACM-1 (a) with delocalised polaronic states and (b) with delocalised polaronic states removed. (c) Nudged elastic band potential energy surface of ACM-1. Diabatic PES i and f corresponds to the initial and the final diabatic PES, respectively.

References

(S1) Oberhofer, H.; Reuter, K.; Blumberger, J. Charge Transport in Molecular Materials: An Assessment of Computational Methods. *Chem. Rev.* **2017**, *117*, 10319–10357.

(S2) Kick, M.; Grosu, C.; Schuderer, M.; Scheurer, C.; Oberhofer, H. Mobile Small Polarons Qualitatively Explain Conductivity in Lithium Titanium Oxide Battery Electrodes. *J. Phys. Chem. Lett.* **2020**, *11*, 2535–2540.
